# Unnatural amino acid incorporation in *E. coli*: current and future applications in the design of therapeutic proteins

**DOI:** 10.3389/fchem.2014.00015

**Published:** 2014-04-01

**Authors:** Kim Wals, Huib Ovaa

**Affiliations:** Division of Cell Biology, Chemical Biology and Drug Innovation, The Netherlands Cancer InstituteAmsterdam, Netherlands

**Keywords:** unnatural amino acid incorporation, *Escherichia coli*, amber codon suppression, release factor 1, therapeutic proteins, antibody conjugates, chemical protein modifications

## Abstract

Unnatural amino acid (UAA) incorporation by amber codon suppression offers scientists a powerful tool to modify the properties of proteins at will. UAA incorporation has been used for a plethora of fundamental research applications and, more recently, also for the selective modification of therapeutic proteins. In this review most recent developments in *Escherichia coli* codon expansion and, unnatural amino acid incorporation are discussed together with some remarkable recent developments in improved efficient UAA incorporation. We focus on the generation of proteins that hold promise for future therapeutic applications that would be impossible to obtain without unnatural amino acid incorporation, including the generation of bi-specific antibodies and antibody drug conjugates.

## Introducing modifications in *Escherichia. coli* produced proteins

*E. coli* is the protein production workhorse of many scientists as *E. coli* is suitable for large scale (industrial) production of many proteins. This prokaryotic workhorse however is not able to incorporate most eukaryotic post-translational modifications (PTMs), such as ubiquitination, glycosylation and phosphorylation, nor is it capable of other eukaryotic maturation processes, and proteolytic protein maturation. In addition, correct disulfide bond formation can be cumbersome. Lipopolysaccharide contaminations can also be troublesome for *E. coli*-produced therapeutic proteins, although sensitive methods for their detection and removal exist (Lopes et al., 2010). Proteins of therapeutic significance, such as antibodies, enzymes and cytokines, commonly carry PTMs and disulfide bonds and often require proteolytic maturation to attain their correctly folded active conformations. Although the majority of therapeutic proteins are produced in eukaryotic and mammalian cell systems, *E. coli* protein expression is in general cheaper, more susceptible to genetic modifications, and versatile with regard to mutant library development. In addition, *E. coli* is fast growing and suitable for industrial scale fermentation (Huang et al., 2012). Moreover, eukaryotic and mammalian cell systems are prone to contamination, often require special growth media and glycosylation systems must be frequently bypassed or disabled in order to produce humanized therapeutic proteins without introducing extra factors that could induce immunogenicity (Hermeling et al., 2004; Kruszewska et al., 2008).

To take the advantages that *E. coli*-mediated expression offers, several strategies have been developed to introduce mammalian protein modifications. If, for example, glycosylation of a recombinant protein is required, specialized *E. coli* strains can be used that are capable of glycosylating proteins. These strains have been developed by transplanting and adapting the *N*-glycosylation system found in *Campylobacter jejuni*, which glycosylates similarly to the eukaryotic glycosylation machinery (Wacker et al., 2002). The humanized glycosylation machinery adapted from *C. jejuni* and other strains are currently under development and may soon provide glycoproteins with control over the specific glycoform that is required in *E. coli* that can be used as therapeutic agents (Schwarz et al., 2010; Terra et al., 2012). Simple eukaryotic hosts, such as *Pichia pastoris*, are more frequently used to generate single glycoforms. The company GlycoFi, for example, uses a series of modified variants of *P. pastoris* to produce antibodies with specific human *N*-glycan structures (Li et al., 2006).

Other strategies are needed to obtain correct disulfide formation of recombinant proteins produced in *E. coli*. An example of a complex therapeutic protein is proinsulin, it consists of two polypeptides containing 3 disulfide bridges. Rudolph et al. showed that a fusion of pro-insulin to the periplasmic *E. coli* protein; disulfide oxidoreductase (DsbA) resulted in a high-yield, correctly folded and bioactive protein produced in *E. coli* (Winter et al., 2001). The periplasm of *E. coli* is the most favorable compartment for disulfide formation since it provides oxidizing conditions and contains proteins like DsbA that can catalyze disulfide bridge formation. Efforts have also been made to express disulfide-rich proteins into the cytoplasm with some success by removing several proteins that balance the redox potential in the cytoplasm of *E. coli*. (Salinas et al., 2011).

These examples show that a number of PTMs can be acquired by clever cloning strategies and by transfer of PTM machineries from other organisms. The introduction of other eukaryotic PMTs, e.g., lysine methylation (Nguyen et al., 2009), acetylation (Neumann et al., 2008, 2009) and ubiquitination (Virdee et al., 2010, 2011) has been achieved by a different approach: *amber codon suppression* to incorporate unnatural amino acids (UAAs) (Noren et al., 1989). This technique allows for the incorporation of a single UAA (i.e., not one of the common 20 amino acids that can be encoded) at a specific site in a protein using a tRNA that recognizes one of the natural stop codons, the so-called “amber” codon. Not only can PTMs be introduced by this technique, but also various chemical handles and groups allowing the post-expression re-design of the properties of proteins. The focus of this review will be on the application of this technique for the design of proteins of therapeutic value. In the first section the amber codon suppression technique including some recently reported developments will be discussed. In the second part current methodologies for modifying the properties of proteins will be discussed as well as the design of specific conjugates.

## Amber codon suppression

**Figure 1:**
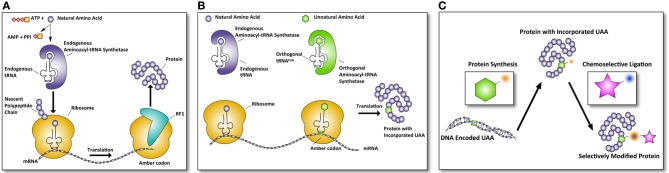
**Incorporation of UAAs into proteins allows selective modifications. (A)** Normal translation is terminated by the recruitment of release factors. In the case of an amber codon, release factor 1 (RF1) is recruited and terminates the translation of the polypeptide rereleasing the newly synthesized protein. **(B)** Amber codon suppression to incorporate unnatural amino acids (UAA) makes use of the amber codon as a coding codon in translation. The complementary amber tRNA^CUA^ is aminoacylated by an orthogonal aminoacyl-tRNA synthetase (aaRS) that is specifically designed to accept only unnatural amino acids. This results in a protein with an UAA incorporated. **(C)** Subsequently, this protein can be selectively modified via chemoselective chemical modification, only reacting with the UAA incorporated in the protein, leaving all other groups in the protein unaffected. In this manner, defined proteins of therapeutic interest can be generated.

The ribosome translates mRNA into a polypeptide by complementing triplet-codons with matching aminoacylated tRNAs. Three of the 64 different triplet codons do not code for an amino acid, but cause recruitment of a release factor resulting in disengagement of the ribosome and termination of the synthesis of the growing polypeptide. These codons are called; ochre (TAA), opal (TGA), and amber (TAG). Of the three stop codons, the amber codon is the least used in *E. coli* (~7%) and rarely terminates essential genes (Nakamura et al., 2000; Xie and Schultz, 2005b). The amber codon triplet in DNA is (TAG), in mRNA (UAG), and the corresponding tRNA anticodon is (CUA). The mRNA triplet UAG of the amber codon, or any other stop codon normally causes the termination of translation by recruitment of one of two release factors, RF1, and RF 2 (see Figure 1A).

Certain species do not use the amber codon as a stop codon, but instead use it to introduce an amino acid at a stop codon. For example *Methanococcus jannaschii* introduces a tyrosine at a UAG codon (Wang et al., 2001). These UAG-tRNAs have been used with great success to introduce unnatural amino acids (UAAs) into proteins in *E. coli* expression systems. In order to achieve this, a series of essential steps had to be taken (Figure 1). The first of these is the evolution of such an orthogonal tRNA and its related aminoacyl-tRNA synthetase (aaRS) so it no longer recognizes a natural amino acid, but instead recognizes a UAA of choice (Normanly et al., 1990). The evolved aaRS needs to be orthogonal to the tRNA loading machinery of the expression host, as otherwise cross-loading of natural amino acids onto the tRNA^CUA^ can occur (see Figure 1B). The first reported aaRS and iso-tRNA pair that was orthogonal to the *E. coli* expression machinery was derived from the aforementioned archaea *Methanococcus jannaschii*, where it normally encodes for a tyrosine residue (Wang et al., 2001). The tRNA^CUA^ pair could be readily mutated to accept an unnatural amino acid, because the aaRS has minimal interaction with the anticodon of its tRNA^CUA^. The minimalist anticodon recognition of the aaRS to its tRNA makes it possible to mutate the aaRS amino acid binding pocket from tyrosine to an UAA with little loss of affinity and aminoacylation efficiency of the aaRS for the tRNA (Steer and Schimmel, 1999). Furthermore, the lack of an editing mechanism capable of deacylating the UAA and high expression levels are the features that have made this pair so successful for *E. coli* (Xie and Schultz, 2005a). This success has been further compounded when the crystal structure of an evolved *M. jannaschii* tyrosyl-RS, the *p*-cyanophenylalanine-specific aaRS, was solved revealing the reason for its atypical polysubstrate (more than 18 UAAs) specificity of this evolved aaRS (Young et al., 2011). This structure has been used for a study that uses molecular modeling and docking to design optimal mutations for a given UAA (Armen et al., 2010).

Takimoto et al. in 2011 published a structure of aaRS for pyrrolysine (Takimoto et al., 2011), which is a second aaRS/tRNA^CUA^ that is often used to incorporate UAAs. This exotic aaRS was found in the archaea *Methanosarcina mazei* and aminoacylates the 22nd genetically encoded natural amino acid pyrrolysine onto a rare tRNA^CUA^ with an amber codon [the 21st genetically encoded amino acid is selenocysteine with a tRNA^UCA^ opal codon (Stadtman, 1996)]. The authors showed that they could evolve the pyrrolysyl-RS on the basis of the crystal structure to accept and aminoacylate the tRNA^CUA^ with UAAs with short aromatic side chains, in contrast to the long aliphatic side chain of the native substrate pyrrolysine.

## Mutant selection with single genetic constructs

**Figure 2:**
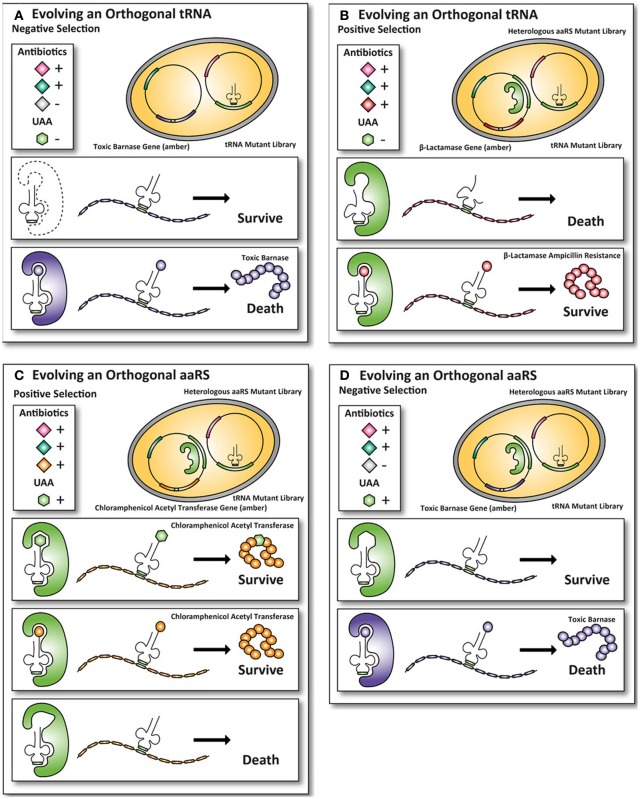
**Classic method for evolving orthogonal aminoacyl-tRNA- synthetases and iso-tRNA pairs for *E. coli.*** To incorporate UAAs in proteins expressed in *E. coli*, an orthogonal aaRS and iso-tRNA pair need to be evolved such that they do not cross-react with the endogenous *E. coli* expression machinery. The orthogonal pair uses an amber stop codon as a coding codon to incorporate an UAA into the protein of interest. Multiple rounds of positive and negative selections are usually carried out to obtain the orthogonality toward the *E. coli* expression machinery. **(A)** First, a tRNA^CUA^ mutant library with an amber codon anticodon is transformed into *E. coli*. To ensure that the endogenous *E. coli* aaRS does not aminoacylates the tRNA^CUA^, an amber-encoded toxic barnase protein is co-expressed. If an endogenous *E. coli* aaRS aminoacylates the tRNA^CUA^, the cell dies because the toxic barnase protein is expressed. **(B)** Next, the tRNAs that are not aminoacylated by the endogenous *E. coli* aaRS survive and are co-expressed with a heterologous aaRS mutant library. In this positive round the cells grow in the presence of the antibiotic ampicillin. If a heterologous aaRS aminoacylate the tRNA^CUA^, the cells survive, because they express the amber-encoded β-lactamase protein, allowing the cells with an aaRS that can aminoacylate the tRNA^CUA^ to survive in the presence of the antibiotic ampicillin. **(C)** To select for mutant aaRS that can aminoacylate the tRNA^CUA^ with an UAA, the UAA is added to the growth medium of *E. coli* and the cells grow in the presence of the antibiotic chloramphenicol. The aaRS that aminoacylate with either the UAA or a natural amino acid survive because an amber-encoded chloramphenicol acetyltransferase is expressed. To select for mutant aaRS that can only aminoacylate with the UAA the mutant library with the aaRS and the tRNA^CAU^ are transferred. **(D)** The cells are now grown in the absence of the UAA. Cells containing mutant aaRS that aminoacylate the tRNA^CUA^ with a natural amino acid die, because they express the amber-encoded toxic barnase protein. Only the UAA specific aaRS and tRNA^CAU^ pairs survive, because they are orthogonal to the *E. coli* expression machinery (Wang and Schultz, 2001; Wang et al., 2001; Xie and Schultz, 2005b).

The classical method to generate UAA-accepting mutants (explained in Figure 2) of orthogonal aaRS and iso-tRNA pairs for *E. coli* (Xie and Schultz, 2005b), as described by P.G. Schultz, is cumbersome as it requires multiple steps of plasmid purification and transformation. (Wang and Schultz, 2001). Extensive efforts have been made to optimize mutant selections that require fewer steps compared to the classic method described in Figure 2 and enable researchers to perform faster selections of desired mutants.

One of these methods was described by Santoro et al. in 2002, where a single genetic construct carrying both the genes for the orthogonal aaRS and iso-tRNA pair from *M. jannaschii* with a chloramphenicol-resistance reporter was used to select highly active aaRS variants in one step (Santoro et al., 2002). In addition, this system contained a T7 RNA polymerase with an amber codon and a green fluorescent protein (GFP) reporter protein construct under the T7 promoter. So only mutants with effective orthogonal aaRS and iso-tRNA pairs could express GFP through T7 promoter induced expression by the amber-encoded T7 RNA polymerase. By making mutations in the amino acid binding site of the aaRS in this genetic construct, libraries of mutants were obtained that efficiently suppressed the amber codon. By FACS-sorting of GFP positive *E. coli* cells from the non-fluorescent cells, multiple rounds of optimizations were easily performed.

A new series of selection vectors was developed by Melancon 3rd et al. They reported a dual positive/negative selection system for the evolution of aaRS with altered specificities in *E. coli* (Melancon and Schultz, 2009). This specific plasmid utilized a chloramphenicol acetyltransferase-uracil phosphoribosyltransferase (UPRT) fusion gene which was used as a dual positive/ negative selection marker. UPRT is an enzyme that can convert 5-fluorouracil to 5-fluorouracil mono phosphate (Rackham and Chin, 2005). The converted 5-fluorouracil monophosphate is an inhibitor for the essential thymidylate synthase. This essential enzyme generates thymidine monophosphate, which is subsequently phosphorylated to thymidine triphosphate for DNA synthesis. In the chloramphenicol transferase part of the fusion protein with UPRT, an amber codon was inserted at a tolerated position. The activity of the amber suppression was selected for in the positive selection step by growing the *E. coli* in the presence of both chloramphenicol and an UAA. Next the negative selection was performed in the presence of 5-fluorouracil.

The most recent improvement to the method for generating amber codon mutants was the development of the plasmid pEVOL (Young et al., 2010). Young et al. developed a system through the use of both constitutive and inducible promoters, based on a plasmid described by Celliti et al. (Cellitti et al., 2008). The constitutive promoter *glnS*' (Plumbridge and Soll, 1987) and the inducible arabinose promoter *araBAD* (Guzman et al., 1995) drove the transcription of two copies of the p-cyanophenylalanine specific aaRS from *M. jannaschii*. (Wang et al., 2001). The advantage of having both a constitutive and an inducible promoter for the aaRS is that there is a basal level present of the aaRS by the constitutive promoter and when protein expression is induced increased levels of the aaRS are obtained by the addition of arabinose. The tRNA^CAU^ was optimized by mutating several positions in the tRNA called the T-stem, and was put under the *proK* promoter of which it is reported to be highly efficient in the transcription of single or multiple copies of the tRNA^CAU^ (Ryu and Schultz, 2006). With the pEVOL vector they efficiently incorporated an UAA in the model protein GroEL which they expressed in approximately 100 mg per liter culture medium.

However, this pEVOL is still a widespread used method to obtain amber codon mutants, this system has now been extended with the addition of a new plasmid-system: pUltra (Chatterjee et al., 2013). This novel dual system allows single- and multiple-UAA mutagenesis in *E. coli* in combination with the previously reported pEVOL amber-suppressor plasmid. The pUltra system encodes both an amber (*M. jannaschii* tyrosyl tRNA/aaRS pairs) and an ochre (*M. barkeri* pyrrolysyl) suppression system. Furthermore, a novel replication origin and antibiotic resistance marker (spectinomycin) allowed an orthogonal use in combination with pEVOL. The described suppression efficiency and the versatility to use multiple UAA orthogonal tRNA-aaRS pairs using this pEVOLV/pUltra system show that the UAA-field is constantly improving and revolutionizing.

## Release factor 1 knockout and amber codon replacement for increased amber codon suppression

A major drawback of amber codon suppression in eukaryotic and prokaryotic cell expression systems is the presence of the amber-recognizing release factor protein 1 (RF1). Prokaryotes terminate translation by RF1 and RF2, where RF1 recognizes the mRNA stop codons UAA (ochre) and UAG (amber), RF2 recognizes UAA (ochre) and UGA (opal) (Scolnick et al., 1968). When the amber codon is reassigned to incorporate an UAA, the tRNACUA is always in competition with RF1, resulting in truncations of the desired protein at the amber position. It has been commonly accepted that both RF1 and RF2 are essential for *E. coli*, and knock-out of one of the RFs is lethal (Gerdes et al., 2003). This lethality is due to a few essential genes with amber codons. However, recent reports describe strains in which the RF1 gene is disabled increasing the amber codon suppression efficiency.

An RF1 knockout was first generated in *E. coli* by Mukai et al., This strain was viable as long as seven essential genes along with the suppressor tRNA were complemented on a plasmid (Mukai et al., 2010). The result was an increasing yield of full-length proteins containing UAAs, because of the lack of translation termination by RF1. D. B. F. Johnson et al. reported the knockout of RF1 by a different approach requiring no addition of any other genes or a suppressor tRNA^CAU^. They created an *E. coli* strain termed JX33 which is RF1 independent, suggesting that RF1 is non-essential in this strain. They complemented these results by expressing GFP in this strain containing up to 10-p-acetylphenylalanine (pActF) moieties into designed GFP reporters. (Johnson et al., 2011). Shortly after these findings D. B. F. Johnson et al. reported that a mutation in RF2 (threonine 246 to alanine) allowed to knockout RF1 in certain *E. coli* strains. RF2 recognizes the stopcodons UAA and UGA. The shared stopcodon UAA between RF1 and RF2 in the *E. coli* genome terminates ~64% of the genes (Nakamura et al., 2000). Johnson et al. hypothesize that the alanine 246 to threonine mutation in RF2 decreases the affinity for UAA. The *E. coli* strains they used to create the RF1 knockout all have an alanine at position 246, suggesting that the A246 in required for efficient termination of the abundant UAA and UGA stop codons in *E. coli*.

The authors also showed that for their *E. coli* strains used, only 7 of the 302 essential genes end with UAG. Additionally, these 7 essential UAG amber-stop codon genes all have an UGA or UAA second stop codon downstream in short distance of their amber-stop codon. If the amber codon is suppressed, the essential genes are “saved” by the second stop codon termination of these proteins by RF2, with only a small number of amino acids added to the essential genes (Johnson et al., 2012).

Next to RF1 knockout, enhanced amber codon suppression could be even further exploited by genomic manipulations of the *E. coli* endogenous genome. As mentioned before the amber codon is the least used in *E. coli* (~7%) and rarely terminates essential genes (Nakamura et al., 2000). Isaacs et al. re-engineered the *E. coli* genome to remove all 314 amber codons and replaced them with ochre codons abolishing the genetic dependence on RF1 (Isaacs et al., 2011).

## Four-base blank codons and evolved ribosomes

The one major limitation to amber codon suppression is that it only allows the replacement with one type of amino acid. In order to encode multiple distinct UAAs into proteins the use of new “blank” codons has been explored. These blank codons require aaRS and iso-tRNA pairs that can decode these blank codons. One example of a further expansion of the genetic code made use of orthogonal aaRS and iso-tRNA pairs that recognize four-base codons. This would in theory provide 256 blank quadruplet codons, and with larger codons even more blank codons are theoretically possible (Anderson et al., 2002; Wang et al., 2012). The use of four-base codons is elegantly applied to Ribo-X, which is an evolved orthogonal ribosome developed by J. Chin and coworkers (Rackham and Chin, 2005). His team demonstrated the synthetic evolution of an orthogonal ribosome, Ribo-X that increased the efficiency of amber codon suppression. They combined Ribo-X with an orthogonal aaRS and iso-tRNA pair from *M. jannaschii* and an orthogonal mRNA. Using this method they substantially increased the efficiency of site specific UAA incorporation in *E. coli*. The Ribo-X discriminates the orthogonal mRNA by a designed sequence that allows only the synthesis of UAA encoded proteins (Rackham and Chin, 2005). They hypothesized that their Ribo-X has less functional interaction with RF1, allowing the tRNA^CAU^ to more efficiently compete for the binding in the presence of an UAG amber mRNA codon (Wang et al., 2007). Although Ribo-X has allowed to incorporate an UAA at two sites with reasonable efficiency (~20% for 2 amber codon sites with Ribo-X and <1% for normal ribosomes), RF1 knockout in the JX33 strain showed higher UAA incorporation of a reporter protein with multiple amber codon sites (Johnson et al., 2011). In 2010 J. Chin and coworkers used their evolved ribosomes (Ribo-X, evolved to Ribo-Q1) to incorporate two UAAs into the protein Calmodulin. Calmodulin fusion protein contained an AGGA codon at position 1 and an amber codon at position 40 of calmodulin. The full length protein was produced with yields up to 0.5 mg per liter culture (Neumann et al., 2010).

## Selective bioorthogonal chemical modification for UAAs

The list of UAAs incorporated into proteins is rapidly growing and other papers review list the growing number of UAAs that can be incorporated in this manner (Budisa, 2004; Hendrickson et al., 2004; Liu and Schultz, 2010; Neumann, 2012). Although the repertoire of UAAs is broad and increasing constantly, in order to use the UAAs for chemical couplings, the reactions should be chemo-selective, bioorthogonal and mild, preserving the fold, and function of the proteins (see Figure 1C). For every new UAA, chemical modifications have to be experimentally determined, fortunately an abundance of invaluable bioorthogonal chemistries for chemoselective protein modifications is described that can help scientists to choose the appropriate reaction for their purpose and protein of interest, (Sharpless, 2002; Prescher and Bertozzi, 2005; Meldal and Tornoe, 2008; Best, 2009; de Graaf et al., 2009; Sletten and Bertozzi, 2009; Lim and Lin, 2010).

## *E. coli* produced therapeutic proteins and amber codon suppression

Despite the numerous technical advances, the main difficulty with UAA incorporation using amber codon suppression is the protein yield. Especially when producing proteins containing multiple UAAs, expression levels are low, which, for now prevents their use in the production of therapeutic proteins. There have been recent advances, however, in the production and use of therapeutic proteins carrying single UAAs, which will be discussed below.

There are numerous examples of therapeutic proteins; insulin for diabetes, human growth hormones for growth deficiencies, antibodies, cytokines and interferons for cancer immunotherapy and several enzymes and blood factors for heart attacks and strokes. Recombinant *E. coli* produced human insulin (Humulin) was the first recombinant human therapeutic protein (Strohl and Knight, 2009). Humulin was developed together by Genentech and Eli Lilly, and first approved for use in 1982 (Strohl and Knight, 2009). Nearly 30% of currently approved recombinant proteins are produced in *E. coli* and the recent advancements are reviewed (Huang et al., 2012) and clinically relevant therapeutic proteins have been reviewed thoroughly elsewhere (Leader et al., 2008).

## PEGylation and UAA modifications of serum proteins

Common features of (recombinant) therapeutic proteins are: a high molecular weight, short half-life and a tendency to become immunogenic to the patient after prolonged use (Hermeling et al., 2004). Another disadvantage can be found in the delivery of these proteins: daily administrations via injections are often necessary to maintain sufficient levels of the protein in the body. In order to overcome these problems therapeutic proteins are frequently modified to enhance their pharmacological properties. The covalent attachment polyethylene glycol chains (PEG) can improve stability of proteins *in vivo* (Pisal et al., 2010; Payne et al., 2011). Chemical strategies to modify these proteins are usually limited to the *N*-terminal amino group, the *C*-terminal carboxylic acid, the ε-amino group of lysine or thiol-groups like cysteine. Modifications at the termini can alter the function of proteins and lysine and cysteine side chain modifications can occur at multiple positions within a protein, making selective modification challenging and frequently yielding heterogeneous products. In addition, the lack of selectivity modifying a protein can lead to loss of biological activity and possibly an increase in immunogenicity (Hermeling et al., 2004). Coupling conditions to connect molecules such as PEG to a protein should be sufficiently mild, to leave the integrity of the protein intact. Whereas exploiting the ε-amino group of lysine to covalently modify it with PEG generally yields heterogeneous products, for homogenous proteins site-specific reactive groups can be inserted into proteins by amber codon suppression. Not only PEG can be introduced in this manner, PTMs from higher eukaryotes, for example sulfonated tyrosine can be introduced by UAA incorporation as well (Liu et al., 2007).

An excellent example of introducing PEG in a site-specific manner is the paper of Deiters et al., where they report a PEGylation methodology based on the site-specific incorporation of *p*-azidophenylalanine in the enzyme superoxide dismutase (Deiters et al., 2004). Using a mild [3 + 2] cycloaddition between the incorporated azido group on the phenylalanine and an alkyne derivative of PEG with copper Cu(I) catalysis they obtained a superoxide dismutase modified with a stable triazole linked PEG chain (Wang et al., 2003) without a significant loss of superoxide dismutase activity (Veronese et al., 2002).

In 2011 Cho et al. further developed PEGylation methodology by site-specific incorporation of PEG in human growth hormone. They incorporated p-acetylphenylalanine at distinct locations in human growth hormone and by the orthogonal chemical reactivity of the *p*-acetyl group of the UAA they formed a covalent oxime bond with an amino-oxy terminated PEG. With this strategy they made 20 different homogeneous forms of PEGylated human growth hormone on a large scale by high density fermentations that have been carried out a 1000 liter scale, yielding 500–800 mg per liter of UAA incorporated human growth hormone. They tested the activity, stability, half-life, and safety of the UAA PEGylated human growth hormone in preclinical rodent studies. The Tyr35 to *p*-acetylphenylalanine mutant form of human growth hormone was tested in growth hormone-deficient adult patients for the tolerability, safety, half-life, and IGF-1 response and showed efficacy and safety that complemented or exceeded daily therapy with human growth hormone produced in mammalian cells (Cho et al., 2011).

Another example of a therapeutic protein modified through amber codon suppression is Hirudin. This protein is an anticoagulant that inhibits thrombin. This therapeutic protein is extracted from leeches and the natural protein contains a sulfonated tyrosine residue. Recombinant *E. coli* expressed hirudin (thus without sulfation of this tyrosine residue) has approximately 10-fold less affinity over the sulfo-hirudin isolated from leeches. By site-specific incorporation of sulfotyrosine, Lui et al. generated a biosynthetic *E. coli*-produced sulfo-hirudin by amber codon suppression. Although the yields were low (5 mg per liter) for the sulfo-hirudin produced by *E. coli* and amber codon suppression they made a co-crystal of sulfo-hirudin and thrombin. Something that was not possible before with the low amounts of hirudin extracted from leeches (Liu et al., 2007).

These examples have shown that be either controlling the PEGylation or by incorporating UAAs in a site-specific manner, improved therapeutic proteins are obtained in a defined and controlled manner which resulted in modified proteins with better therapeutic value. (de Oliveira et al., 1999; Jung et al., 2011).

## *E. coli* produced antibodies: modifications and advancements

Antibodies are well recognized as proteins of therapeutic value and are important therapeutically for a wide range of diseases, such as autoimmunity and cancer therapy. As such there are many efforts ongoing in order to improve the effector functions of therapeutic antibodies. Several modified antibodies are used in the clinic or evaluated in clinical trials. Although none of the reported antibodies are capable of curing cancer as a single agent, (Chames and Baty, 2009) specific antibodies can improve the clinical outcome of cancer, autoimmune disease, transplant rejection or can bind toxins (e.g., snake venom toxins). The use of therapeutic antibodies is reviewed elsewhere (Leader et al., 2008).

Extensive research has been conducted in order to produce humanized N297 *N*-glycosylated IgG antibodies. This *N*-linked glycan affects the therapeutic properties of antibodies by recruiting innate immunity effector cells and serum proteins, such as complement system proteins. Recruitment of these effectors can lead to targeted cell death, which is a desirable property in cancer immunotherapy. Many efforts have been made and several systems have been developed [mammalian, non-mammalian expression systems, like the GlycoFl-engineered *P. pastoris* from GlycoFi, mentioned before (Li et al., 2006)] to obtain glycosylated IgG antibodies. Although *N*-linked glycosylated N297 is the natural glycosylated residue found in human IgG antibodies, unglycosylated antibodies can be produced in *E. coli* and some of these recombinant antibodies are in clinical trials. So far, all clinically approved therapeutic antibodies have been produced in fused mouse myeloma cells or Chinese hamster ovarian (CHO) cells (Jung et al., 2011). Despite the fact that no approved therapeutic antibodies are from *E. coli* origin, *E. coli* still remains an attractive system to produce non-glycosylated antibodies and continuing efforts are made to scale up the production including methods that use heat-inducible expression (Bayly et al., 2002) and metabolic engineering (Makino et al., 2011).

Another example of ongoing developments includes the production of Iclonals. These *E. coli* produced and refolded IgG antibodies and antibody-toxin fusion proteins show similar binding affinities compared to mammalian cell culture antibodies. Using the Iclonal technique, 50 mg of pure, refolded antibody was obtained from 1 liter shake flask cultures by Hakim et al. (Hakim and Benhar, 2009). The benefit of this system is the speed; antibodies were obtained in these amounts within in 8–9 days, compared to the multi-week process for a mammalian expression system. In the next example we discuss how UAA incorporation can provide more flexibility and versatility and can be used to create therapeutic bispecific antibodies and antibody-drug conjugates.

## Bispecific antibodies

**Figure 3:**
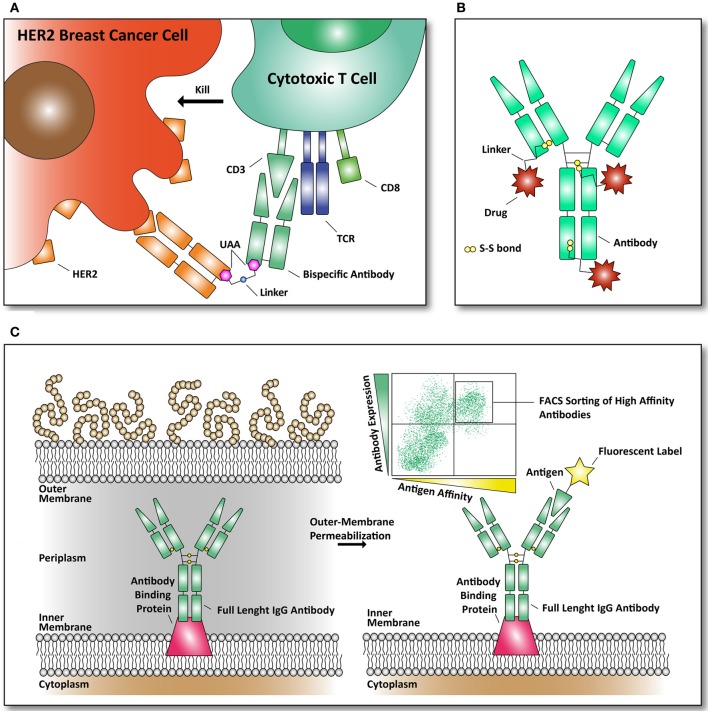
**Examples of reported antibodies and screening methods with pharmacological relevance. (A)** Bispecific antibody against HER2 and CD3 linked together through an UAA. The basic principle of a bispecific antibody is that it can bring two targets together. Our immune system can eradicate cancer cells, through recognition by killer immune cells that destroy them (Chames and Baty, 2009). As depicted, a bispecific antibody can bring cytotoxic T cells in contact with the cell surface receptor HER2, frequently found over-expressed in breast cancer cells (Kim et al., 2012). **(B)** Structural overview of an antibody drug conjugate (ADC). Through specific targeting of a plasma membrane marker (example: fibronectin, a marker of tumor angiogenesis) a bound drug (example: cytotoxic drug) can be released to the targeted marker (Bernardes et al., 2012). Until now, only natural amino acids, like cysteine, are used to conjugate cytotoxic drugs to the antibodies through thiol-bonds. **(C)** Antibody library formats in *E. coli* are used to screen for antibodies with improved affinity for their antigen. The antibodies are expressed in *E. coli* and displayed on the inner-membrane. By outer-membrane permeabilization the antibodies are exposed and suitable for FACS-mediated sorting with fluorescently labeled antigens (Mazor et al., 2010).

One of the most recent examples of using amber codon suppression to incorporate UAAs in an *E. coli* produced antibody is the synthesis of a bispecific antibody as recently described by Schultz and coworkers. An anti-HER2 /anti-CD3 bispecific antibody was constructed, using the fragment antigen binding domains of both antibodies (see Figure 3A) (Kim et al., 2012). The CD3 protein is associated with the T cell receptor (TCR) of mature CD8+ T cells. These cytotoxic CD8+ T cells can kill both virus-infected cells and tumor cells. Their TCR and CD8+ receptors have evolved to detect intracellular pathogens and tumor cells by recognition of peptides, derived from viruses and tumor proteins, displayed on the cell surface by MHC class I molecules. Once presented on the cell surface in MHC complexes, T-cells can recognize them and kill the target-cell by releasing cytotoxins to kill the infected or tumor target-cell. As a result of the efforts of Schultz and coworkers a remarkable molecule was constructed that brings T cells to HER2-expressing (breast cancer) cells with *in vitro* effector-cell mediated cytotoxicity at picomolar concentrations. Thus, this approach brings a T cell in close proximity to a HER2-expressing breast cancer cells to form a cytotoxic synapse. Tumors frequently escape immune surveillance by down-regulating MHC class I antigen presentation, preventing cytotoxic CD8+ T cells from recognition and clearance (Seliger, 2008).

For the construction of the bispecific HER2-CD3 bispecific antibody, a similar approach as described in the paper from Schultz was used where p-acetylphenylalanine was incorporated at distinct locations in human growth hormone to couple a PEG moeity (Cho et al., 2011). In this study p-acetylphenylalanine was incorporated in mutant Fab fragments of an antibody that recognizes HER2 (Herceptin, Cho et al., 2003) and (CD3 UCHT1, Zhu and Carter, 1995) by amber codon suppression. Then the two antibody fragments were selectively coupled by a stable oxime bond to bifunctional linkers with an alkoxy-amine on one terminus and an azide or cyclooctyne group at the other. Next they coupled the two bifunctional linkers to obtain the bispecific antibody by a copper-free [3 + 2] Huisgen cycloaddition (Jewett and Bertozzi, 2010).

The use of an UAA provided in the given example can certainly be applied to other antibody fragments. Linker fragments can be modified and optimized in order to obtain the right flexibility and affinity needed for the bispecific antibody. In addition, UAA incorporation can provide more specificity and flexibility, with regard to standard bispecific antibody generation. In the review from Chames and Baty most recent clinical trials involving the use of bispecific antibodies (and other antibody-formats) are listed and the authors describe the success of these therapeutic antibodies in great detail (Chames and Baty, 2009). The previous example of the HER2-CD3 bispecific antibody is one of many combinations that are possible. Most bispecific antibodies use the reactivity of lysine and cysteine leading to heterogeneous products. The advantage of site-specific incorporation of UAAs compatible with crosslinking is that they are bioorthogonal and yield a single defined product, which is difficult to obtain through standard amino acid coupling chemistries.

## Antibody drug conjugates

A different use of modified antibodies comes in the form of antibody-drug conjugates (ADCs). Here a drug can be targeted to a receptor that is up-regulated in various malignant cells. For example, the human epidermal growth factor receptor 2 (HER2) is frequently found over-expressed in breast cancer cells and provides a target that can be used for ADCs. In addition, the transferring receptor and the epidermal growth factor receptor are often used targets to combat cancer (Daniels et al., 2006; Dhomen et al., 2012). We point the reader to two reviews (Chari, 2008; Senter, 2009) that list the majority of ADCs, used linkers and their bound drug targets. The ADC approach of delivery makes it possible to use potent, but highly toxic drugs that cannot be applied systemically because they cause side effects due to off-targeted effects (i.e., side-effects of the drug affecting healthy cells). By using ADCs potent drugs can accumulate at the tumor site, avoiding the systemic toxicity and side effects. There are several mechanisms in how an ADC can release the drug to the target and the mode of action depends on the specificity of the antibody. The first mechanism is continuous internalization. Once the ADC is bound to its target it will get internalized eventually and the drug bound to the antibody can be released in various ways, including proteolysis and pH-mediated drug release. Other mechanisms include ADC accumulation, before internalization and in other cases the drug can be released at the site of interest through a different environment (Chari, 2008). For example, as a tumor grows, tumor cell lysis mediated glutathione and cysteine release to the tumor microenvironment allows the drug to be released from the ADC in a target-specific manner (Bernardes et al., 2012).

Most ADCs are based on the reactivity of thiol groups present as cysteine (see Figure 3B). A recent publication from Axup et al. has applied unnatural amino acid incorporation to site-specifically make an ADC (Axup et al., 2012). Here they have designed an HER-2 Fab fragment in *E. coli* and a full length IgG in mammalian CHO cells with high yields of 400 mg/ml and 300 mg/mL, respectively using high-density fermenters. *Via* the site-specifically incorporated p-acetylphenylalanine they coupled the microtubule toxin auritstatin through a stable oxime-ligation. Both the ADC Fab and ADC IgG full length were converted with a yield of >95%, making this approach very suitable for preparing homogeneous, well-defined ADC's.

This example demonstrates that the use of UAA incorporation can benefit the preparation of a site-specific ADC. There have been reports of near-homogeneous ADC preparations and site-specific coupling a cysteine in ADCs with great efficacy and potent activities *in vivo* (Junutula et al., 2008; Bernardes et al., 2012). This near-homogeneous approached used the reactive thiol in cysteine for coupling maleimide-drugs. These thiol-based reactions can influence the ADC stability and therapeutic activity (Shen et al., 2012). Difficulties for maleimide chemistry involve disulfide bond reduction reoxidation procedures. Here, UAA incorporation provides more gentle coupling chemistries and offers a more versatile tool to define the position of the drug and the use of cleavable and non-cleavable linkers.

## Antibody libraries produced in *E. coli*

In general *E. coli*–based antibody libraries are not as successful as other formats reported, like yeast- or phage-antibody display libraries (Boder et al., 2000). *E. coli* is limited by its antibody expression, secretion and folding. An important factor for an antibody screening-library is the accessibility and cellular localization of the antibody (Hoogenboom, 2005). Nevertheless, to identify antibodies with improved affinity for their antigen, mutant-based libraries in *E. coli* can be used. In the review by Hoogenboom the most important library formats are discussed and the various antibody and antibody-like proteins that can be obtained from these libraries is demonstrated (Hoogenboom, 2005).

To overcome this major drawback of *E. coli* based antibody-libraries, Mazor et al. developed an elegant technique to obtain full length antibodies from *E. coli*. They selected for full-length IgG antibodies displayed on *E. coli* by assembling these antibodies in the periplasm of *E. coli*. The authors expressed an antibody binding protein in the inner-membrane of *E. coli* to bind the antibody produced and by subsequent outer-membrane permeabilization the antibodies were assessable to fluorescently labeled antigens. By binding these fluorescently labeled antigens to the outer-membrane permeabilized *E. coli* cells, they could select for strong-binding IgGs in multiple rounds of FACS (see Figure 3C). The obtained antibodies were reported to be well expressed in *E. coli* and had nanomolar affinity (Mazor et al., 2007, 2008).

Two papers that use UAA incorporation in combination with library display in *E. coli* come from the Schultz lab and these efforts resulted in significantly improved antibody affinities upon UAA incorporation. In the first examples the authors genetically incorporated sulfotyrosine into gp120 antibodies by amber codon suppression. It is of note that a specific antibody found in human HIV patients contains two sulfotyrosine residues and participate in the strong interaction for the gp120 envelope glycoprotein of HIV (Choe et al., 2003). Based on this PTM from this antibody, the authors made a phage-display library of amber codon mutant antibodies and showed that it is feasible to combine a library screening approach with UAA incorporation (Liu et al., 2008, 2009a). In a more recent example from the Schultz lab a genetically encoded p-boronophenylalanine, was incorporated in order to trap diols that are present in carbohydrates through the selective formation of boronic acid esters. The single-chain variable-chain antibody phage library was then used to select for antibodies that can trap glucamine. By performing multiple enrichment rounds they could obtain about 80% of clones containing the UAA after three rounds of selection (Liu et al., 2009b).

Besides altering UAA incorporated antibodies, there have been widespread efforts in the protein engineering field to manipulate antibody fragments, design protein scaffolds and create non-Ig-binding proteins like affibodies. These non-Ig-binding proteins are designed to bind protein targets and are greatly suitable for randomized libraries of mutants. Some of these novel engineered therapeutic proteins have progressed into the clinic for targeted cancer therapy, regulated drug delivery, and *in vivo* imaging. Affibodies and other new alternate antibody scaffolds which could benefit from UAA incorporation have been reviewed in detail (Banta et al., 2013).

## Future applications of UAA incorporation

Numerous future applications can be thought of by combing existing techniques and UAA incorporation for protein modification. Next, we will briefly discuss these applications of the future that may hold promise for the development of novel therapeutic modalities.

**Figure 4:**
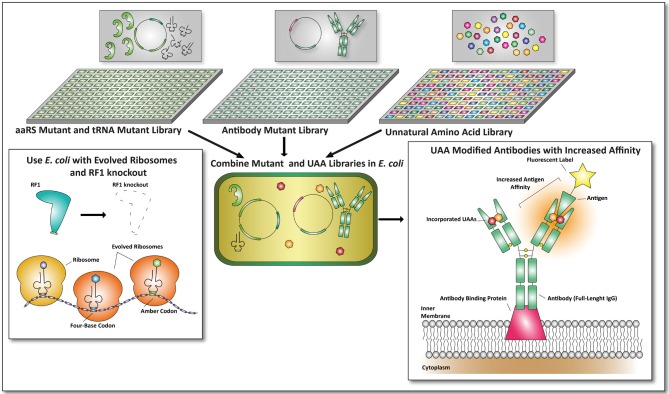
**Future applications of unnatural amino acid incorporation for the development of proteins with therapeutic value.** In this example the antibody format described by Mazor et al. is used to combine UAA incorporation with *E. coli*-based screening formats (Mazor et al., 2010). An aaRS mutant and tRNA mutant library can be cross-combined with an antibody mutant library. Together with unnatural amino acid libraries, screens can be performed on viable mutants expressing antibodies with improved affinities toward a fluorescent antigen. To make this screening process more efficient, an *E. coli* strain with an RF1 knock-out (Johnson et al., 2012) and evolved ribosomes (Wang et al., 2012) can be used. By combining this, amber codon suppression and four-base codon suppression could yield promising and undiscovered therapeutic proteins.

Since it is possible to combine UAA incorporation with library based selection procedures, a next generation of antibodies, protein scaffolds, and non-Ig-binding proteins could be obtained by making use of the techniques described here and by combining them. This is not limited to the creation of bispecific antibodies as described earlier, or the use of a single defined UAA to screen for improved antibodies. Multiple UAA incorporation can be used to incorporate different UAAs to improve the affinity of antibodies toward their antigen. In a similar fashion orthogonal aaRS and iso-tRNA libraries can be co-transformed into *E. coli* to obtain mutant antibodies with incorporated UAAs, provided that appropriate selection procedures are in place (see Figure 4).

By combining *E. coli* mutant libraries with a library of UAAs new and possibly highly potent antibodies or other proteins could be engineered. Since all the techniques are currently available, next-generation therapeutic proteins can be identified exploiting the advances of UAA incorporation in more straightforward manners. Not only increased target affinities of proteins with therapeutic value through the incorporation of UAAs can be expected, also metabolic stabilities can be altered in this manner as proteins can now be designed to become resistant to proteolysis or early secretion.

Although UAA incorporation has enormous promise, it is also in its infancy and significant hurdles are currently being addressed. The technique is currently far from routine, however in the near future it may be part of the toolbox available for drug design. Engineering the *E. coli* to increase the efficiency of amber codon suppression and UAA incorporation are in currently being intensely investigated as evidenced by the recent publication of the RF1 knockout *E. coli* strain JX33, that allows the incorporation of multiple UAAs in one protein and genome wide amber codon replacement (Isaacs et al., 2011; Johnson et al., 2011). In addition the generation of single genetic constructs for orthogonal aaRS and iso-tRNA pairs has made it easier to mutate and screen for efficient amber codon suppression. The introduction of evolved ribosomes and four-base codons, UAA incorporation has enabled the incorporation of more than one defined UAA per protein, although increasing the efficiency of incorporation of multiple UAAs is still problematic and more technological developments are needed in order to make it applicable for future therapeutic applications.

### Conflict of interest statement

The authors declare that the research was conducted in the absence of any commercial or financial relationships that could be construed as a potential conflict of interest.

## Abbreviations

UAA: Unnatural Amino Acid
PTM: Post-Translational Modification
DsbA: Disulfide Oxidoreductase
aaRS: aminoacyl-tRNA Synthetase
tRNA: transfer RNA
mRNA: messenger RNA
RF: Release Factor
GFP: Green Fluorescent Protein
FACS: Fluorescence Assisted Cell Sorting
UPRT: Uracil Phosphoribosyl transferase
IPTG: Isopropyl β-D-1-thiogalactopyranoside
PEG: polyethylene glycol
TCR: T Cell Receptor
ADC: Antibody-Drug Conjugate.
